# Development and Evaluation of a Pilot Nurse Case Management Model to Address Multidrug-Resistant Tuberculosis (MDR-TB) and HIV in South Africa

**DOI:** 10.1371/journal.pone.0111702

**Published:** 2014-11-18

**Authors:** Jason E. Farley, Ana M. Kelly, Katrina Reiser, Maria Brown, Joan Kub, Jeane G. Davis, Louise Walshe, Martie Van der Walt

**Affiliations:** 1 School of Nursing, Johns Hopkins University, Baltimore, Maryland, United States of America; 2 College of Nursing, Michigan State University, East Lansing, Michigan, United States of America; 3 Bloomberg School of Public Health, Johns Hopkins University, Baltimore, Maryland, United States of America; 4 Tuberculosis Epidemiology and Intervention Research Unit, Medical Research Council, Pretoria, South Africa; University of Aveiro, Portugal

## Abstract

**Setting:**

Multidrug-resistant tuberculosis (MDR-TB) unit in KwaZulu-Natal, South Africa.

**Objective:**

To develop and evaluate a nurse case management model and intervention using the tenets of the Chronic Care Model to manage treatment for MDR-TB patients with a high prevalence of human immunodeficiency virus (HIV) co-infection.

**Design:**

A quasi-experimental pilot programme utilizing a nurse case manager to manage care for 40 hospitalized MDR-TB patients, 70% HIV co-infected, during the intensive phase of MDR-TB treatment. Patients were followed for six months to compare proximal outcomes identified in the model between the pre- and post-intervention period.

**Results:**

The greatest percent differences between baseline and six-month MDR-TB proximal outcomes were seen in the following three areas: baseline symptom evaluation on treatment initiation (95% improvement), baseline and monthly laboratory evaluations completed per guidelines (75% improvement), and adverse drug reactions acted upon by medical and/or nursing intervention (75% improvement).

**Conclusion:**

Improvements were identified in guideline-based treatment and monitoring of adverse drug reactions following implementation of the nurse case management intervention. Further study is required to determine if the intervention introduced in this model will ultimately result in improvements in final MDR-TB treatment outcomes.

## Introduction

The convergence of the *Mycobacterium tuberculosis* (TB) and human immunodeficiency virus/acquired immunodeficiency syndrome (HIV/AIDS) epidemics in southern Africa has led to many challenges for both providers and the healthcare system [1]. South Africa has the highest incidence rate of TB per capita [2] and TB is the leading cause of mortality in HIV-infected patients [3]. Drug-resistant TB remains a growing threat to public health. Multidrug-resistant TB (MDR-TB), defined as resistance to isoniazid and rifampicin, now accounts for 3.6% of newly-diagnosed TB cases and 20% of all previously-treated TB cases globally [2].

MDR-TB treatment is complex, requiring a minimum six-month intensive phase of treatment with an injectable aminoglycoside, followed by an 18- to 24-month continuation phase. In addition, MDR-TB treatment is costly, with an average per patient cost of $6772 [4]. In South Africa, more than 70% of TB patients are HIV co-infected [5]. Co-infection further complicates the MDR-TB treatment course through the risk of overlapping drug toxicities [6], [7], fragmentation of services [8], and increased financial strain on health systems [9]. Further, compromised immunity from HIV infection presents a greater challenge in the diagnosis of TB and results in more rapid HIV/TB disease progression [3].

MDR-TB treatment success has been shown to be significantly lower among MDR-TB patients co-infected with HIV who are not on antiretroviral therapy (ART), with data from multiple South African cohorts demonstrating earlier mortality [10], [11] and MDR-TB success rates of less than 50% (with success defined as cure or completion of treatment) [12], [13]. The administration of ART during MDR-TB treatment has demonstrated improved MDR-TB outcomes for co-infected patients in southern Africa, further supporting the need to integrate care [11], [14]. The implementation of an integrated HIV and MDR-TB patient-centered, evidence-based approach has the potential to improve treatment outcomes [15]. We proposed the use of a nurse case manager to achieve these goals.

Nurse case managers in HIV care have been shown to increase access to care and provide safe, ongoing, and effective disease management in South Africa [16]. Nurse case management (NCM) is a patient-centered, collaborative approach in which a registered nurse facilitates and coordinates treatment plans to ensure that timely, evidence-based care is given to improve treatment outcomes [17]. This paper describes the development and implementation of a programmatically based pilot intervention utilizing a NCM model for MDR-TB and HIV care. The NCM model was developed using the tenets of the Chronic Care Model (CCM) [18]. The CCM utilizes a systems approach that encourages and sustains productive interactions between clinicians and patients and includes five dimensions: delivery system design, decision support for clinicians, clinical information systems, self-management support, and community resources [19], with this pilot investigation focusing on the first two domains. To further customize our intervention to the given setting, we used the PRECEDE-PROCEED planning tool for designing, implementing and evaluating programmatic change to select the six proximal outcomes to test during our NCM intervention [20], [21]. In 2012, we investigated the use of a nurse case manager to improve the outcomes presented in our model for MDR-TB patients with a high prevalence of HIV co-infection. We hypothesized that a nurse case manager would improve MDR-TB and HIV guideline adherence by addressing six proximal outcomes selected from the South African MDR-TB or HIV treatment guidelines, which have been shown to improve MDR-TB treatment outcomes. This article presents the improvements seen in the proximal outcomes at an inpatient MDR-TB unit in South Africa over a six-month period.

## Methods: Model Development

### Modification of the Chronic Care Model using the PRECEDE-PROCEED approach

Treatment and care of MDR-TB patients, with or without HIV, requires sustained interventions over a minimum two-year period. To modify the CCM for this setting and healthcare context, we utilized the nine-phase PRECEDE-PROCEED model in the tailoring and evaluation of the intervention in this vulnerable population. The final four phases included the implementation of the intervention and the evaluation of the process, impact and outcomes, presented in the results section.

Phase 1 Social Assessment: To assess the feasibility of rolling out the NCM model for MDR-TB/HIV intervention on a provincial scale, we conducted two focus groups (n = 10 per group) with nurses working in MDR-TB wards in KwaZulu-Natal (KZN) Province. The nurses focused on defining the social problems that have an impact on MDR-TB treatment outcomes. These health system and patient level themes are presented in Table 1.

**Table 1:** Health system and patient level themes from HCW focus groups related to MDR-TB treatment outcomes in South Africa.

Health System Level	Patient Level
Under-resourced treatment setting	Sociodemographic characteristics, including poverty and rural setting
Cultural and language barriers between physicians and patients	HIV status complicates treatment
Limited inpatient bed capacity resulting in long pre-treatment wait lists	Prolonged time from MDR-TB diagnosis to start of treatment
Limited MDR-TB/HIV knowledge of treatment guidelines in both inpatient and primary health centers (PHC)	Perception that MDR-TB was a result of prior poor treatment adherence
Lack of MDR-TB/HIV treatment integration	Stigma
	Gender roles resulted in imbalance of care

Phase 2 and 3 Epidemiologic, Behavioural and Environmental Assessments: Further assessments were conducted through both a comprehensive review of published literature, as well as primary country level data collection. Relevant World Health Organization (WHO) reports on drug-resistant TB [6], [22] were reviewed and we held multiple discussions with key stakeholders within the National Department of Health (DOH) to assess the epidemiology of MDR-TB/HIV in South Africa. In conducting our behavioural and environmental assessments, we conducted a country level evaluation of MDR-TB treatment facilities to determine the available infrastructure [23] and assessed health care worker (HCW) fears working with MDR-TB patients [24]. As part of the environmental assessment, we also evaluated HCW knowledge of MDR-TB programmes [23]. We compiled these findings in a SWOT analysis (strengths, weaknesses, opportunities and threats) of the current MDR-TB and HIV treatment model (Table 2).

**Table 2:** Strengths, Weaknesses, Opportunities and Threats (SWOT) analysis of current MDR-TB and HIV treatment model.

	HELPFUL	HARMFUL
**INTERNAL**	**Strengths**	**Weaknesses**
	Evidence-based MDR-TB and HIV guidelines	Lack of human resources
	Integrated HIV and TB goals in National Strategic Plan	Lack of HCW training
	Political leadership for improving treatment outcomes at national level	Poor infection control and HCW fear of infection
	Rapid diagnostic testing availability	High burden/prevalence of HIV/MDR-TB co-infection
	Decentralized and community-based management (including home visitation)	Low inpatient bed capacity
	Local partnerships/collaboration	Poor transportation infrastructure to access MDR-TB treatment facilities
		Inconsistency across programmes on guideline implementation
		Poor treatment incentive structure (grants spent on non-health related family needs)
**EXTERNAL**	**Opportunities**	**Threats**
	Partnership/collaboration between research and clinical team well established	Decentralized and community-based management (potential for fragmentation)
	Emerging research on TB/HIV treatment integration and improved outcomes	Lack of inter-professional collaboration among HCWs
	Prioritization of MDR-TB treatment in national spotlight	Low health literacy
		Poor ART management
		Inter-provincial migration

**Figure 1:**
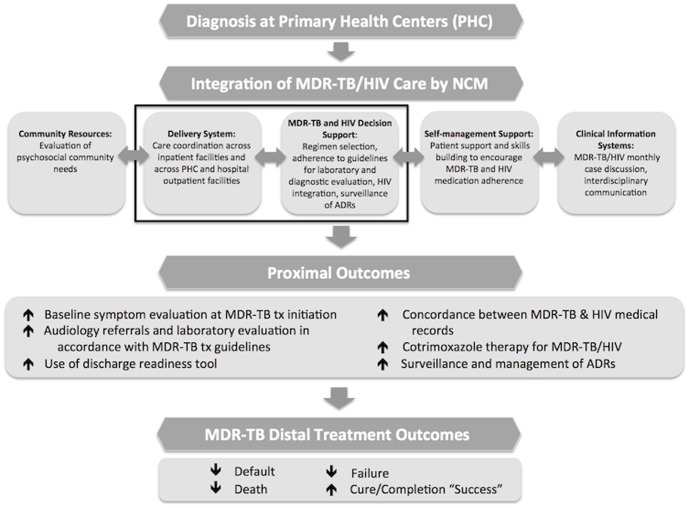
Conceptual framework for NCM elements to improve MDR-TB proximal outcomes. Domains of Delivery System and MDR-TB and HIV Decision Support were tested in this study. (tx = treatment).

Phase 4 Educational and Ecological Assessment: Our team evaluated the predisposing, enabling, and reinforcing factors associated with MDR-TB treatment that could affect guideline adherence and HIV integration. To evaluate predisposing factors (i.e. individual beliefs) we looked at HCW attitudes regarding guideline adherence. To evaluate enabling factors (i.e. availability of resources) we conducted an evaluation of HIV integration within the MDR-TB unit. Lastly, to evaluate reinforcing factors (i.e. community attitudes) we conducted the aforementioned focus groups. We determined which factors were most likely to result in sustained behaviour change and presented them as our proximal outcomes in the final model (Figure 1). We predicted that the six proximal outcomes in our NCM model would lead to improved distal MDR-TB treatment outcomes.

Phase 5 Administrative and Policy Assessment: Our team conducted a programmatic review of the South African DOH and WHO guidelines on MDR-TB and HIV treatment in the determination of which proximal outcomes to test in the NCM model. At the time of our pilot evaluation, the National DOH adopted a strategy of 100% guideline adherence as a goal of the country's National Strategic Plan for HIV and TB (2012–2016) [5], further supporting the need for an integrated model of care at the administrative level.

### Final NCM Pilot Model Implementation (Phase 6)

The PRECEDE-PROCEED planning process led to two main changes in the final intervention implementation: a Zulu-speaking nurse case manager would be employed to address the need for greater linguistic and cultural competency and all proximal outcomes in the model would come directly from the MDR-TB and/or HIV treatment guidelines. The training of this nurse case manager was modified to incorporate the findings of our predisposing, enabling and reinforcing factors. We presented the refined model to local DOH authorities in Ugu District in KZN province in 2012. Implementation of the NCM intervention occurred in a single MDR-TB center in order to evaluate the feasibility and process outcomes of implementing and expanding the model throughout Ugu District. As nurse case managers are a new cadre of HCWs in MDR-TB treatment and care, our pilot evaluation focused on only two elements of the overall CCM: a) delivery system and b) decision support (Figure 1). Our focus group data (Table 1) identified rich information about patient level variables. These data informed our decision to select a member of the local community as the case manager to address the language barrier. For the remainder of this pilot study, we choose to focus on six outcomes at the health system level.

### Delivery System Proximal Outcomes

In the CCM, the delivery system domain focuses on linkage and access to care. Farley et al. [12] found on average, a delay of more than two months from culture-based MDR-TB diagnosis to treatment initiation in a sample of 757 patients. This was likely attributed to issues with diagnostic delay and limited inpatient bed availability. In the pilot setting for this intervention, the nurse case manager was instructed to focus on: 1) discharge planning through the use of a tool to determine readiness of admitted patients for discharge; and 2) concordance between the MDR-TB and HIV medical records on ART regimen.

### Decision Support Proximal Outcomes

Comprehensive treatment guidelines for the management of MDR-TB are available in South Africa and follow the evidence-based guidelines of the WHO [25]. Although nurse case managers do not prescribe, they provide decision support to the prescriber to assist in clinical care for the complex MDR-TB/HIV patient through the implementation of treatment guidelines. To influence the proximal outcomes related to decision support, the nurse case manager was instructed to implement the following: 3) scheduling and follow through of required laboratory and diagnostic testing (e.g. audiometry); 4) baseline symptom screening at the time of admission with a head-to-toe physical exam and symptom checklist; 5) initiation of cotrimoxazole preventative therapy for HIV co-infected patients, as appropriate; and 6) active surveillance through patient interview for any adverse drug reactions (ADRs).

## Methods: Data Collection

We conducted a quasi-experimental pilot intervention to test the NCM model. The pilot included the hiring and training of one full-time Zulu-, Xhosa- and English-speaking nurse case manager to improve and evaluate the six proximal outcomes identified in the model. This intervention was conducted among 40 patients admitted to the MDR-TB center in Ugu District. We compared pre- and post-intervention proximal outcomes for patients hospitalized over the intensive phase of MDR-TB treatment within the CCM domains of delivery system and decision support.

### Ethical Approval

Approval was granted for this programme-based intervention by the Medical Research Council (MRC) of South Africa, along with the research committee of the KZN DOH. Approval was sought from the Johns Hopkins University Institutional Review Board, but the design was deemed a quality improvement programme and therefore exempt from human subjects review and not requiring informed consent. Since the intervention was deemed to present no more than minimal risk of harm for study participants among ethical review boards (MRC and KZN DOH), both review boards approved the protocol and granted a waiver of documented informed consent. Focus groups were utilized in the development of the pilot intervention. Nursing participants at the end of MDR-TB capacity development workshops were given the opportunity to participate in these focus groups. Before participation, they were informed of the topic of discussion, the risks and benefits of participation, and the manner in which confidentiality would be maintained. Their continued presence upon closing of the workshops was considered confirmation of their consent. This was approved by the aforementioned ethical review boards.

## Results

Forty hospitalized MDR-TB participants, 50% female, were enrolled. The median age was 47 years (range 16–78) and all participants were Zulu-speaking, black South African. Forty-eight percent of the sample reported having no steady employment. Seventy percent (n = 28) were HIV-infected with 75% (n = 21) receiving ART at baseline. The same 40 participants were followed for the entire six-month intervention period during their inpatient hospital stay. All participants were included upon initiation of the intensive phase of their MDR-TB treatment, which constitutes daily injections administered by a member of the healthcare team. The nurse case manger worked at the MDR-TB unit an average of 20 hours per week to deliver the intervention over the six-month period.

### Process and Impact Evaluation (Phase 7 and 8)

To evaluate the implementation process, weekly calls and/or site visits were held between the study coordinator and the nurse case manager, as well as monthly calls with the principal investigator to ensure adherence to the intervention. For impact evaluation, we observed participant response to the intervention. Participants actively sought out the nurse case manager to discuss their care with 100% participation across both male and female participants with no loss to follow-up during the six-month intervention period. They repeatedly requested that the nurse advocate on their behalf to the medical officer for health concerns, side effect management and for assistance with psychosocial matters at home.

### Proximal Outcomes for NCM Delivery System Interventions (Phase 9)

In the pre-intervention period, the nurse case manager identified that patients awaiting admission were not admitted based on acuity and that inpatients received no discharge planning. The nurse case manager pilot-tested the use of a discharge readiness tool to increase bed availability across the 40 participants as a process outcome measure. At the time of the intervention, the nurse case manager was not able to initiate early discharge through the use of the tool, as community-based support for aminoglycoside injections was not yet in place. The minimum length of the intensive phase according to the South African guidelines is six months, so participants remained hospitalized for the full duration of the intervention. Regarding concordance between the MDR-TB and HIV medical records, the nurse case manager identified 44% of documented ART regimens were discordant between the medical records at baseline. Post-intervention concordance was identified in 100% of medical records (Table 3).

**Table 3:** Changes between baseline and six-month pilot study intervention period for selected NCM intervention domains.

Domains	Interventions to Improve Proximal Outcomes in NCM Model	Changes Between Baseline and Six-Month Pilot Study Intervention Period (n = 40)		
		Pre-Intervention Period (Baseline)	Post-Intervention Period (Six-Month)	Percent Difference
**Health Care Delivery System**	**HIV/MDR-TB medication integration between health records**			
	Concordance on ART regimen and dosing between HIV and MDR-TB medical charts	56%	100%	44%
**MDR-TB and HIV Decision Support**	**Adherence to South African HIV and MDR-TB guidelines**			
	a. Referral/completion of audiology screening per guidelines	30%	100%	70%
	b. Monthly laboratory evaluations completed per guidelines	10%	85%	75%
	c. Receiving cotrimoxazole preventative therapy (n = 28 with HIV co-infection)	64%	88%	24%
	d. Baseline symptom evaluation on MDR-TB treatment initiation	5%	100%	95%
	**Active surveillance of adverse drug reactions (ADRs)** *			
	ADRs acted upon by medical or nursing intervention	25%	100%	75%

* Pre-intervention period used passive, patient self-report of ADRs; intervention period used active surveillance for ADRs.

### Proximal Outcomes for NCM Decision Support Interventions (Phase 9)

The nurse case manger identified major deficits across all areas of MDR-TB guideline adherence in the pre-intervention period and was able to demonstrate improvement across each of the proximal outcomes. At baseline, 30% of patients received audiology referrals according to the guidelines and 10% of patients received monthly laboratory evaluation. Post-intervention, this improved by 70% and 75%, respectively (Table 3). Baseline symptom evaluation increased by 95% and cotrimoxazole preventative therapy increased by 24%. Full implementation of active surveillance for ADRs during the intervention phase resulted in a 35% increase in the number of events per patient, from 2.6 to 3.5. Additionally, a weekly goal sheet was implemented for all admitted patients in the intervention period, resulting in a 75% increase in actions taken to mitigate ADRs.

## Discussion

In this novel study, we developed and piloted a nurse case manager intervention. Following implementation, we identified improvements in adherence to MDR-TB and HIV treatment guidelines in both the delivery and decision support domains of the CCM. Prior research has demonstrated how a NCM approach leads to significant improvements in disease management and outcomes such as improved treatment adherence, decreased psychosocial distress and reduced morbidity and mortality in diseases such as heart failure [26], [27], diabetes [28], HIV [16], [29]–[31], and drug susceptible TB [32], [33]. Nyamathi et al. conducted a randomized controlled trial of NCM in the United States (U.S.) among homeless HIV-infected persons with latent TB showing that nurse case managers increased the rate of completion of isoniazid preventative therapy (OR: 3.26; 95% confidence interval, 2.13–4.99) [33]. The same impact of NCM was identified by Lin and colleagues who demonstrated a significantly higher rate of successful TB treatment compared to patients without NCM in a hospital setting (240/277, 86.6% vs. 67/92, 72.8%; p = 0.002) [32]. Data from these studies support the use of a multifaceted, patient-centered NCM intervention to improve treatment outcomes. The present study adds to these data by supporting the opportunity for NCM in the MDR-TB patient population. Our results also support the use of the CCM in a resource-limited setting. Previous studies have also utilized the CCM among marginalized HIV-infected populations to improve the ongoing management of the disease, but this approach has primarily been tested in resource-rich settings [34].

The grounding of this intervention within the CCM, with the use of the PRECEDE-PROCEED model, is hypothesized to provide small health system level (or proximal) changes that will ultimately lead to improved distal MDR-TB treatment outcomes in a high HIV-burdened population. A U.S.-Zambian collaborative study highlighted the effectiveness of a similar approach to prevent HIV in a resource-limited setting. In this study, Jones et al. began by choosing an evidence-based intervention, utilized an established theoretical model to translate the intervention to a community level setting, and pilot-tested an adapted form of the intervention with initial support at the provincial level. This ultimately led to programme rollout in 61 clinics with a 75–80% delivery rate of the intervention [35]. Likewise, we hypothesized that the proximal outcomes addressed in our NCM model will lead to improved distal MDR-TB treatment outcomes.

While the findings of this pilot study demonstrate improvements in guideline-based care, the study was based on only one trained nurse case manager at a single hospital, testing a small number of proximal outcomes. It remains unclear whether the use of nurse case managers will be feasible and effective in other MDR-TB settings. Our study included the tailoring of a culturally appropriate intervention designed to include a nurse case manager who was from the surrounding area and who spoke the local languages. This aspect of the intervention was not directly measured and the study team is unable to determine the impact of this approach on the proximal outcomes, but we hypothesize that cultural competency may have contributed to improved outcomes. Although we cannot determine the level of impact linguistic and cultural tailoring had for patient participation and retention, our proximal outcomes demonstrate that participants clearly engaged with the nurse case manager for assistance with their care coordination. This positive response warrants the need to empirically test the effect of these components of the intervention in future research. We were also unable to evaluate all domains of the CCM due to limitations in pilot funding, coupled with a desire to test specific aspects related to HCW adherence to guidelines. Overall, testing only two concepts of the model does limit the generalizability of the findings. We were also unable to test with a comparison group and the quasi-experimental design used does not allow for the establishment of a causal relationship. This study was conducted within an inpatient population. At the time of the intervention in 2012, all MDR-TB patients in Ugu District were still being hospitalized for the intensive phase of treatment. Currently in 2014, a greater number of patients are being managed in the community. A community-based setting may present additional challenges, as well as additional health system and economic considerations. Future research will include testing all domains of the CCM within our refined NCM model. This pilot has informed the development and implementation of a cluster-randomized trial comparing nurse case management to standard of care.

## Conclusions

As MDR-TB care continues to be decentralized to the community in South Africa, the NCM model may provide an effective method for integrating the complex care of the MDR-TB/HIV co-infected patient. Potential challenges to implementing this model throughout the South African MDR-TB programme include the feasibility of introducing a new cadre of HCWs and the potential cost implications. Despite these challenges, the demonstrated improvements in MDR-TB/HIV guideline adherence suggest the NCM model as a promising means of ultimately improving MDR-TB treatment outcomes. In addition, such a model may offer a novel method for meeting the South Africa DOH recommendation to increase MDR-TB/HIV guideline adherence.
